# High Frequency Stimulation of the Subthalamic Nucleus Leads to Presynaptic GABA(B)-Dependent Depression of Subthalamo-Nigral Afferents

**DOI:** 10.1371/journal.pone.0082191

**Published:** 2013-12-23

**Authors:** Anton Dvorzhak, Christoph Gertler, Daniel Harnack, Rosemarie Grantyn

**Affiliations:** 1 Cluster of Excellence NeuroCure, Department of Experimental Neurology, University Medicine Charité, Berlin, Germany; 2 Department of Experimental Neurology, University Medicine Charité, Berlin, Germany; Universidade Federal do ABC, Brazil

## Abstract

Patients with akinesia benefit from chronic high frequency stimulation (HFS) of the subthalamic nucleus (STN). Among the mechanisms contributing to the therapeutic success of HFS-STN might be a suppression of activity in the output region of the basal ganglia. Indeed, recordings in the *substantia nigra pars reticulata* (SNr) of fully adult mice revealed that HFS-STN consistently produced a reduction of compound glutamatergic excitatory postsynaptic currents at a time when the tetrodotoxin-sensitive components of the local field potentials had already recovered after the high frequency activation. These observations suggest that HFS-STN not only alters action potential conduction on the way towards the SNr but also modifies synaptic transmission within the SNr. A classical conditioning-test paradigm was then designed to better separate the causes from the indicators of synaptic depression. A bipolar platinum-iridium macroelectrode delivered conditioning HFS trains to a larger group of fibers in the STN, while a separate high-ohmic glass micropipette in the rostral SNr provided test stimuli at minimal intensity to single fibers. The conditioning-test interval was set to 100 ms, i.e. the time required to recover the excitability of subthalamo-nigral axons after HFS-STN. The continuity of STN axons passing from the conditioning to the test sites was examined by an action potential occlusion test. About two thirds of the subthalamo-nigral afferents were occlusion-negative, i.e. they were not among the fibers directly activated by the conditioning STN stimulation. Nonetheless, occlusion-negative afferents exhibited signs of presynaptic depression that could be eliminated by blocking GABA(B) receptors with CGP55845 (1 µM). Further analysis of single fiber-activated responses supported the proposal that the heterosynaptic depression of synaptic glutamate release during and after HFS-STN is mainly caused by the tonic release of GABA from co-activated striato-nigral afferents to the SNr. This mechanism would be consistent with a gain-of-function hypothesis of DBS.

## Introduction

“Deep Brain Stimulation” (DBS) is a relatively new approach in the therapy of cognitive, motor or emotional disorders [1]–[5]. In humans with idiopathic Parkinson syndrom [6]–[10] and in dopamine-deficient rodents [11], [12], continuous high frequency stimulation of the subthalamic nucleus (HFS-STN) was shown to alleviate akinesia. In search of an explanation for this beneficial effect three putative mechanisms have received particular attention: A) Rescue of dopamine signaling [13]–[20], B) Modification of cortical information processing through retrograde signals delivered from the STN to the motor cortex via axons of the hyperdirect pathway [11], [12], [21] and C) HFS-induced changes in the balance of excitation and inhibition in the output nuclei of the basal ganglia (BG) [22]–[25]. All three mechanisms are mutually not exclusive and could combine to facilitate movement initiation (see [26] for more).

In non-human primates akinesia has been reduced by surgical lesion of the STN [27]. Parkinsonian individuals were found to exhibit an increased firing rate in the STN [28], and they benefited from pharmacological inactivation of neuronal activity in the STN [29]. Considering that all STN neurons are glutamatergic [30] and belong to the indirect as well as hyperdirect [31] pathway to the BG output nuclei, one can predict that silencing the subthalamo-nigral pathway would relieve the motor thalamus from tonic GABAergic inhibition and thereby increase motor activity [32], [33].

Initially the beneficial effect of HFS-STN was attributed to a functional deafferentation of the GB output nuclei, consistent with the observation that HFS-STN reduces the firing rate in the SNr [22], [24], [34]. One result in support of this hypothesis is the nearly complete depression of the pharmacologically isolated fiber volleys (FV) in the SNr and the entopeduncular nucleus soon after the start of HFS-STN [35].

However, depending on the stimulus intensities applied and the activity used as an indicator of functional deafferentation, the final results of HFS-STN may also depend on the state of the GABAergic afferents in the SNr. These afferents are likely to be co-activated. In rodents, GABAergic axons originating in the *Globus pallidus* (the equivalent to the *Globus pallidus externus* in primates) pass through the STN, while GABAergic axons from the striatum bypass the STN at some distance to its ventral margin [23]. Multi-unit co-activation of glutamatergic and GABAergic afferents by a relatively large metal electrode may shift the excitation-inhibition balance in the SNr to variable degree. Recordings of inhibitory postsynaptic currents (IPSCs) performed in slices from juvenile rats [36] and mice [37] already hinted the possibility that striato-nigral afferents might be more resistant to depression than pallido-nigral afferents.

To better understand the cellular mechanisms of HFS-STN, it is desirable to perform experiments where the effects of multi-unit HFS are analyzed along with those of single-unit HFS in functionally defined axons impinging onto single SNr neurons. This can be done by applying the method of extracellular minimal stimulation [38] through a high resistance glass micropipette. In our lab, this method was initially used in cultures where unitary activation could be verified on the basis of fluorescence labeling of single axons [39] or by monitoring the induced calcium elevations in attached synaptic terminals [40]. Later on, this approach was adapted to implement a “blind” search for unitary responses in slices, including preparations from fully adult mice [41]. The pre- and postsynaptic indicators of synaptic efficacy were similar to those obtained with neuron pairs. However, in cases when the somata of the coupled neurons are widely separated and a cell-specific reporter is not yet available, unitary extracellular stimulation becomes the method of choice.

We shall present evidence suggesting that the artificial electrical stimulation of the STN enables a mechanism of cellular interaction that may not exist under condition of physiological signal transmission in the basal ganglia. In support of this gain-of-function hypothesis HFS-STN was found to tonically activate the release of GABA, presumably from striato-nigral afferents. Activation of GABA(B) receptors then enabled a form of heterosynaptic depression at the glutamatergic subthalamo-nigral terminals. This depression lasts longer than the depression of axonal excitability and is therefore relevant for subthalamo-nigral afferents with partially preserved/recovered transmitter release function.

## Methods

### Ethical statement

The present experiments were performed in fully adult (315 to 565 days) C57Bl6 mice either provided by the Charles River GmbH Germany or the Jackson Labs (Maine, USA). Every precaution was taken to minimize stress for and the number of animals used in each series of experiments. The work described here has been carried out in accordance with the EU Directive *2010/63/EU* for animal experiments and comply with the requirements for manuscripts submitted to Biomedical journals. The study was registered at the Office of Health Protection and Technical Safety of the regional government Berlin (Landesamt für Arbeitsschutz, Gesundheitsschutz und Technische Sicherheit Berlin, T0448/12) and specifically approved by the Institutional Animal Care and Use Committee of the University Medicine (Charité).

### Slice preparation

For slice preparation the mice were anesthetized by inhalation of a mixture of isoflurane and carbogen (95% O_2_ and 5% CO_2_). The isoflurane concentration was adjusted to prevent spinal reflexes. The mice were then transcardially perfused with 60 ml of ice-cold (∼4°C) saline containing (in mM): 125 choline chloride, 2 KCl, 1.25 NaH_2_PO_4_, 25 NaHCO_3_, 10 glucose, 0.5 CaCl_2_, 7 MgCl_2_, 0.001 MK-801, 0.5 pyruvic acid, 0.005 glutathione, 2.8 ascorbic acid (pH 7.25, 305 mOsmol/l). The brain was removed quickly (∼1 min), separated into two hemispheres and transferred into ice-cold oxygenated saline. To preserve the basal ganglia circuits [42] 300 µm sections were cut at an angle of 10 degrees off the sagittal plane using a vibrating microtome (Integraslice 7550PSDS, Campden Instruments Ltd., Loughborough, UK). After cutting, the sections were then maintained for at least 1 h at 24°C in artificial cerebrospinal fluid (ACSF) that contained (in mM): 125 NaCl, 2 KCl, 1.25 NaH_2_PO_4_, 25 NaHCO_3_, 10 glucose, 2 CaCl_2_, and 1 MgCl_2_ (pH 7.35, 300 mOsmol/l).

For electrophysiological tests, the slices were placed into a recording chamber, maintained under constant flow of oxygenated ACSF (volume 0.4 ml, flow rate 1.5 ml min^−1^) and regularly inspected using a movable Zeiss microscope (Axioscope 2 FS plus, Zeiss, Oberkochen, Germany) equipped with a 20×1.3 NA water immersion objective (Olympus Europa Holding GmbH, Hamburg, Germany) and a camera for infrared videomicroscopy (Till Photonics, Munich, Germany. During the recordings temperature was set to 26–27°C. Preceding experiments at various maintenance temperature (range 23–30°C) showed that under the given conditions slices from animals >1 year were best preserved at this temperature, the quality criterion being the level of neuronal membrane potentials at break-in, in addition to the appearance of the slices.

### Field potential recording

**Figure 1:**
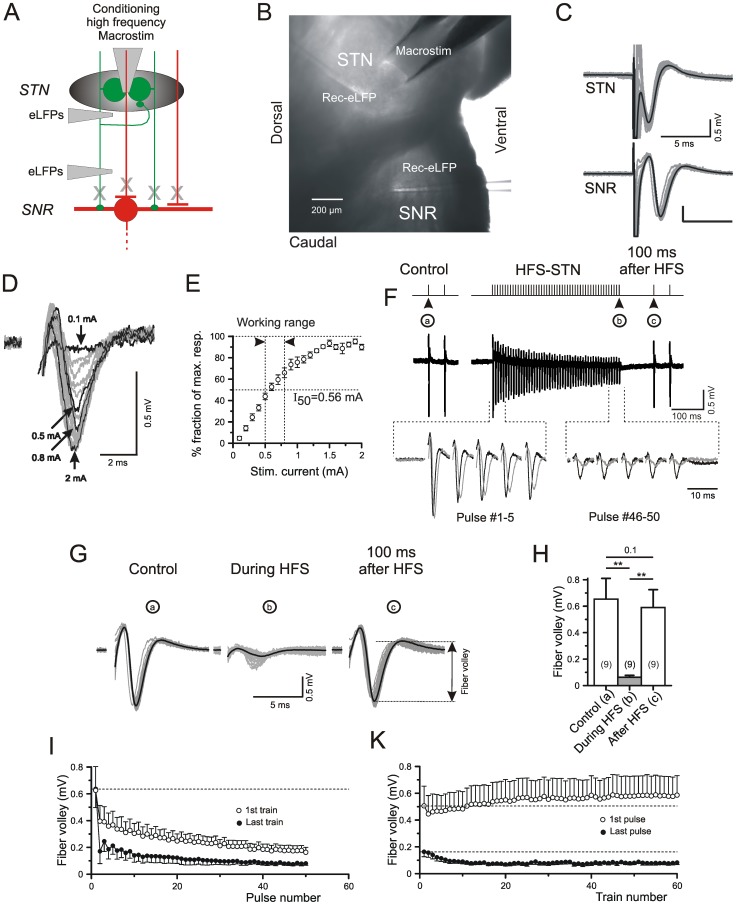
Local field potentials in the SNr after low and high frequency stimulation of the STN. A) Scheme of experiment. B) Image of the slice showing the electrode positions for the experiment illustrated in C. C) Individual (grey) and average (black) traces of FVs in gabazine and DNQX+APV. D) Specimen records of FVs in the SNr at various stimulus intensities. E) Dependency of FV amplitude on stimulus intensities. F) Control response before HFS-STN and single conditioning-test trial. Please note that both the conditioning HFS stimuli and the low frequency test stimuli were delivered through the same bipolar macroelectrode in the STN. (a–c) refer to pulses inducing the FV shown in (G). The traces in the bottom of (F) correspond to the first and the last 5 stimuli within an HFS train. In this case traces in black represent responses during the first HFS train, traces in gray - responses during the last HFS train in a series of 60 HFS trials. G) Individual (gray) and averaged (black) FVs recorded before, during and after HFS-STN, as indicated in (F). H) Quantification of results from 9 slices/9 mice. I,K) Dynamics of FV amplitude within one HFS-STN train (I) and during train repetitions at a rate of 6/s (K). Note that the 1st response in each train recovers fully (I) or even displays slight potentiation with train repetition (K) while the last responses only exhibit depression.

Local field potentials (LFPs) were recorded with electrodes pulled from thick-walled borosilicate glass capillaries (1B150F-4, WPI, Sarasota, USA) and filled with ACSF (resistance 0.8–1 MOhm). The pipettes were placed in the SNr and occasionally also in the STN as shown in Fig. 1B. The signals were amplified, low-pass filtered at 3 kHz, and recorded using a home-made differential amplifier (courtesy B. Schacht, Institute of Neurophysiology, Charité, Berlin), an ITC-16 AD/DA board (Instrutech, New York, USA) and the software TIDA 4.11 (HEKA Elektronik, Lambrecht, Germany). To isolate the fiber volley (FV) component of the LFPs, the GABA(A) and ionotropic glutamate receptors were blocked. After the recording, the FV was also blocked using tetrodotoxin (TTX 1 µM, Alomone Labs, Jerusalem, Israel). The differential traces recorded from the SNr had a time to peak of 3.47±0.22 ms (n = 9, Fig. 1C). In the experiments with HFS-STN FV amplitudes were determined as the voltage difference between the negative peak at ∼3.5 ms and the following positive deflection, as illustrated in Fig. 1G. Only records with a stable baseline and reproducible FV of at least 0.5 mV were used for analysis.

### Whole cell recording

Postsynaptic currents and action potentials were recorded in the whole cell voltage clamp mode. If not mentioned otherwise, the intra-pipette solution contained (in mM): 150 potassium gluconate, 5 NaCl, 0.5 CaCl_2_, 5 EGTA, 25 HEPES, 2 MgATP, 0.3 GTP, pH was set to 7.2 with KOH. The pipette resistance ranged from 3 to 6 MOhm. The experimentally determined reversal potential of GABA(A)-induced chloride currents (E_Cl_) was = −100 mV. The holding potential was set to −60 mV (glutamatergic currents) or to −50 mV (GABAergic currents). The signals were acquired at a sampling rate of 30 kHz using an EPC-8 amplifier connected to the 16-bit AD/DA board. Liquid junction potentials were not corrected. Only recordings with a series resistance below 30 MOhm were accepted (in typical cases series resistance amounted to 15–20 MOhm). Series resistance compensation was not applied. Access resistance was controlled by regularly (1/6 s) applying hyperpolarizing pulses of 10 mV. Cells exhibiting more than 20% changes in the access resistance during an experiment were discarded. Whole cell capacitance and input resistance values were determined by fitting a mono-exponential function to the currents induced by 10 mV hyperpolarizing pulses or single fiber stimulation. The time constants of uIPSC decay were determined by an automatized evaluation routine fitting a monoexponential function to the IPSC decay.

### Electrical stimulation

**Figure 2:**
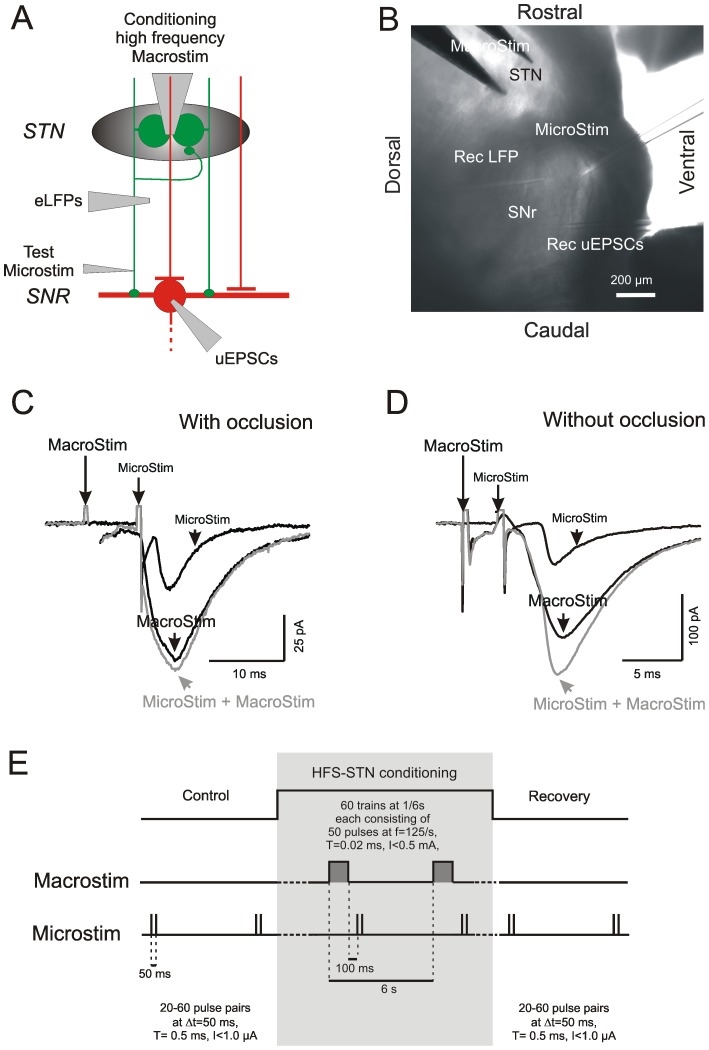
Scheme of occlusion test and conditioning experiment. A) Experimental arrangement showing the involved cellular elements, their connections and sites of stimulation/recording. Green: glutamatergic elements. Red: GABAergic elements. “MacroStim” - bipolar platinum iridium macroelectrode for multi-unit STN activation. “Microstim” - glass pipettes for activation if single axons. B) Image of a sagittal slice comprising the STN and the SNr. Note tip positions of the stimulating and recording pipettes. C,D) Occlusion test to determine whether the unit selected for Microstim belonged to the axonal pool activated by Macrostim. Full algebraic summation of the compound response to Macrostim and the unitary response to Microstim indicates that there is no continuity of the test fiber into the STN (C). Accordingly, full summation is taken as evidence that the fiber selected for Microstim was not occlusion-positive into the STN (D). E) Pulse patterns during conditioning Macrostim in the STN and test Microstim of a single STN fiber in the SNr. Each experimental session consisted of three parts: Control – a pair Microstim pulses at an interstimulus interval of 50 ms, HFS-STN – combination of HFS Macrostim trains followed at an interval of 100 ms by a pair of Microstim pulses, and Recovery – same as control. The repetition rate of the trials was 1/6 s.

The arrangement of the pipettes for stimulation and recording are given for each type of experiment. Details of the pulse pattern are illustrated in Fig. 2E. For multi-unit stimulation (abbreviated as “Macrostim”) bipolar platinum-iridium electrodes were placed into the center of the STN. The electrodes had tip distance of 250 µm and a resistance of 1 MOhm. They were purchased from Science Products GmbH, Hofheim am Taunus, Germany (PI2ST31.0A10). In each slice, the intensity of Macrostim was adjusted to the half maximal current value (EC50) eliciting the FV in the SNr (Fig. 1D,E). A more than 20% increase of the EC50 was regarded as evidence for tissue deterioration at the stimulation site, and the slices were discarded. In experiments with HFS-STN current intensities ranged from 500 to 800 µA at a pulse duration of 0.02 ms.

The PSCs elicited by single-fiber stimulation are referred to as unitary PSCs (uEPSCs, uIPSCs). For the purpose of single-unit activation in the SNr (referred to as “Microstim”) we used ACSF-filled glass micropipettes with a resistance of about 10 MOhm. For Microstim purposes rectangular pulses with 0.5 ms duration were delivered through an optically isolated constant current source (Isolator-11, Axon Instruments, CA, USA). As single fiber activation was performed blindly, it was necessary to explore at any given site a wide range of current intensities. The criteria used to accept a Microstim site are laid down in [41].

The principal stimulation pattern used for the pairing of multi-unit HFS-STN with single-unit stimulation in the SNr is illustrated in Fig. 2E. Each experimental session consisted of three parts (“Control”, “HFS-STN conditioning” and “Recovery”). In all three parts of a conditioning experiment, test pulses were delivered as pairs with an interstimulus interval of 50 ms. The HFS-STN trains consisted of 50 pulses delivered at a frequency of 125/s. The interval between the HFS train and the first test stimulus was 100 ms. This principal pattern was used in all experiments, although the site of HFS varied with the specific aims.

### Solutions and chemicals

The glutamatergic nature of ion currents was verified by applying the AMPA receptor blocker 6,7-dinitroquinoxaline-2,3-dione (DNQX, 10 µM) and the NMDA receptor blocker DL-2-amino-5-phosphonopentanoic acid (APV, 50 µM). Similarly, the GABA(A) receptor blocker 2-(3-Carboxypropyl)-3-amino-6-(4 methoxyphenyl)pyridazinium bromide (gabazine, 15 µM) was used to block GABAergic currents. To test for involvement of GABA(B) receptors, (2*S*)-3-(1*S*)-1-(3,4-dichlorophenyl)ethyl[amino-2-hydroxypropyl](phenylmethyl)phosphinic acid (CGP55845) was applied at a concentration of 1 µM. All these compounds were obtained from Tocris (Bristol, UK). Other chemicals were purchased from Sigma-Aldrich (Munich, Germany).

### Data evaluation and statistics

The data was evaluated off-line using TIDA 4.11 (HEKA Elektronik, Lambrecht, Germany), Origin 4.10 (Microcal Software, Inc., Northampton, MA) and SPSS 12.0 (SPSS GmbH Software, Munich, Germany). EPSC amplitudes are given as absolute values or normalized to the control average obtained for each particular test. The success rate of the unitary EPSC (s.r.) was defined as s.r. = 100%-failure rate of the responses to the first in a pair of test pulses. The analysis of fluctuating EPSCs was performed as described before [43]. Briefly, the paired pulse ratio (PPR) was defined as PSC2/PSC1, and the coefficient of variation of uEPSC amplitudes (CV) is the standard deviation divided by the mean of the uEPSC sample. Changes in the PPR and the CV are commonly attributed to changes in the probability of vesicle exocytosis [43].

The results are presented as mean ± S.E.M. In all figures the error bars indicate S.E.M. Differences between means were tested for significance using Student's *t*-test or ANOVA, unless otherwise stated. The asterisks indicate the following: * - p<0.05, ** - p<0.01, and *** - p<0.001.

## Results

### The fiber volley indicates HFS-induced depression of action potential generation in the subthalamo-nigral pathway

LFPs were elicited by multi-unit activation via bipolar platinum-iridium electrodes in the STN (“Macrostim”) and recorded in the STN and SNr using ACSF-filled glass pipettes as described in Methods. By sequential application of blockers of glutamate and GABA(A) receptors, the respective neuronal and non-neuronal responses to electrical stimulation were eliminated, leaving a short-latency negative deflection (see Fig. 1C). The latter was eliminated by the Na channel blocker TTX and therefore unequivocally identified as equivalent to the compound voltage produced by the generation of local action potentials (APs). Since the pioneer studies of Eccles and colleagues in the nineteen fifties [44], AP-related field potential components were described and analyzed in numerous single cell studies of the brain and spinal cord. They have served to indicate the arrival of action potentials to the target area innervated by a given set of afferents (for instance [45]) or the presynaptic effects of a drug (for instance [46]). Most frequently, the TTX-sensitive LFP component has been referred to as “afferent volley” or “fibre/fiber volley”. In the following text we apply the latter term to make clear that our results obtained with field potential recording only apply to the AP-related/TTX-sensitive component of the LFP.

Fig. 1 provides a scheme of experiment (A) and an image with the positions of stimulating and recording electrodes (B). Examples of FVs in the STN and in the SNr are given in Fig. 1C. The respective peak latencies were 1.21±0.17 ms (n = 8) and 3.48±0.22 (n = 9) ms. Based on this time difference and the distance between the tips of the recording electrodes, we obtained an average conduction velocity of 0.24±0.02 (n = 6) m/s. Although we had good reason to assume that the FVs reflect AP generation in a mixture of different types of fibers, including GABAergic afferents originating in the *Globus pallidus*, the size of the FV proved to be a useful means to predict the preservation of glutamatergic afferents originating in the STN. We requested a FV in the SNr of at least 0.5 mV to proceed with a given slice. In most cases amplitudes were larger than that (Fig. 1D) rendering half-effective current intensities (EC50) in the range of 0.5 to 0.8 mA. The experiment described in Fig. 1F,G was performed with a stimulation intensity of 0.6 mA.

To characterize the dynamics of AP suppression and recovery during and after multi-unit HFS-STN, FVs were elicited by a pair of test stimuli at an interstimulus interval of 50 ms and recorded in the absence and presence of a preceding HFS train. The amplitude changes reported in Fig. 1 are based on comparison of the responses to the first test pulse in the pair (“a” in Control, “c” after HFS). The degree of depression during HFS was determined on the basis of the FV elicited by the last pulse of the train (“b”). Fig. 1G shows the respective specimen traces at larger scale. Fig. 1H-K summarizes the results from 9 different slices/animals. The experiments show a major depression of the FV at the end of the HFS (−89±3%, n = 9, Table 1). However, 100 ms after the HFS train, AP generation recovered to control level. In repeated trains the first responses remained stable (Fig. 1I,K) while the following pulses exhibited increasing depression until a steady level was reached after about 10 trials with HFS.

**Table 1:** Effects of multi-unit and single-unit HFS on glutamatergic unitary synaptic responses in the SNr.

HFS type	Parameter	Control		HFS				
		Average	±SE	Average	±SE	N	%Change	p
**Multi-unit**	FV in STN (µV)	835,08	185,43	861,25	285,79	8	**none**	ns
	FV in SNr (µV)	673,51	154,31	589,44	135,59	9	**none**	ns
**Multi-unit**	uEPSC occlusion-positive							
	Amplitude (pA)	23,26	3,15	5,88	1,14	9	**−75**	<0.001
	PPR	0,84	0,09	1,36	0,20	9	**63**	0,022
	CV (%)	53,97	6,07	179,24	22,18	9	**232**	<0.001
	Success rate (%)	86,53	3,57	33,60	6,75	9	**−61**	<0.001
**Multi-unit**	uEPSC occlusion-negative							
	Amplitude (pA)	46,27	7,58	20,10	4,03	27	**−57**	<0.001
	PPR	0,86	0,06	1,30	0,12	27	**52**	<0.001
	CV (%)	29,64	5,31	78,00	24,09	27	**163**	0,035
	Success rate (%)	89,86	1,87	56,97	5,92	27	**−37**	<0.001
**Multi-unit**	uEPSC occlusion-negative							
	Failures excluded							
	Amplitude (pA)	42,07	8,18	28,11	5,07	15	**−33**	0,002
	PPR	0,83	0,06	1,04	0,06	15	**25**	0,002
	CV (%)	36,67	2,72	38,46	2,72	15	**none**	ns
**Multi-unit**	uEPSC occlusion-negative							
	GABA(B) block							
	Amplitude (pA)	55,66	14,32	37,56	7,50	11	**−33**	0,070
	PPR	0,95	0,10	1,07	0,15	11	**12**	ns
	CV (%)	0,51	0,07	0,84	0,29	11	**63**	ns
	Success rate (%)	89,54	3,75	78,63	8,64	11	**−12**	ns
**Single-unit**	uEPSC							
	Amplitude (pA)	68,69	11,86	26,51	5,28	9	**−61**	0,001
	PPR	0,91	0,09	1,09	0,06	8	**20**	0,033
	CV (%)	41,09	5,29	61,02	5,76	8	**49**	0,007
	Success rate (%)	93,89	2,63	58,89	10,79	9	**−37**	0,011

Recovery kinetics often provides a useful criterion for separating processes of different nature. If applicable, one may select a point in time when one process has ended while the other has not. The above results suggest that 100 ms were sufficient for recovery from AP depression. We therefore used this interval to test for AP- independent effects of HFS-STN and first of all asked whether the glutamatergic subthalamo-nigral synaptic responses were also recovered by this moment of time.

### Electrical back-tracing of individual glutamatergic afferents into the fiber pool activated by STN macrostimulation

The above question could be answered by analyzing the nigral responses to stimulation of single glutamatergic afferents. However, when applying such approach to 400 µm slices, one has to deal with several constraints, among them the fact that some of the subthalamo-nigral axons responding to single fiber stimulation close to the postsynaptic neuron (“Microstim”) may not be part of the multi-unit pool activated by Macrostim in the STN. A fiber activated in the STN may not reach the site of microstimulation in the SNr and, *vice versa*, a glutamatergic fiber monosynaptically connecting to the recorded SNr neuron might be interrupted or otherwise damaged upstream of the Microstim site.

A classical occlusion test, as used in collision experiments to study peripheral nerve branching (e.g. [47], [48]), served the aim of electrical back-tracing the tested axons to the site of HFS-STN. Fig. 2A and B illustrate the experimental scheme and the position of the stimulating and recording electrodes. If the monosynaptic EPSCs in response to Macrostim and Microstim lacked linear summation, the respective afferent was considered to belong to the fiber pool activated upstream by Macrostim in the STN and to connect downstream directly to the recorded SNr neuron (Fig. 2C). In contrast, if the responses exhibited full summation, the fiber was either not within the pool of neurons affected by Macrostim or disconnected between the site of Macrostim and Microstim (Fig. 2D). The interval required to place AP generation into the refractory period after Macrostim was based on the conduction velocity derived from the peak time differences in the FV records from the STN and the SNr. The percentage of glutamatergic unitary afferents that were occlusion-positive, i.e. belonging to the pool activated by HFS-STN conditioning, was 9/25 (36%).

### Conditioning high frequency STN stimulation is suppressive for occlusion-positive as well as occlusion-negative subthalamo-nigral unitary connections

**Figure 3:**
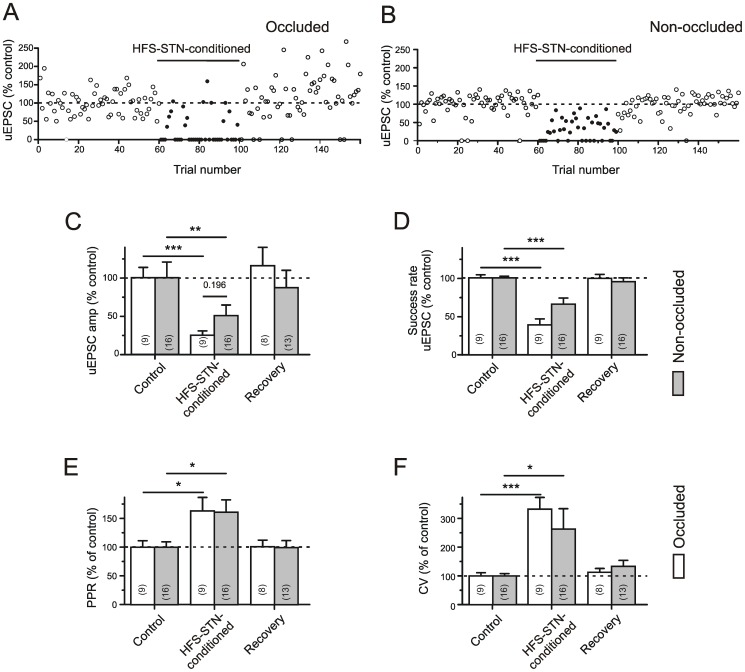
Synaptic depression of unitary EPSCs by conditioning multi-unit HFS-STN. Comparison of occlusion-positive and occlusion-negative afferents. GABA(A) receptors were blocked with gabazine (15 µM). A) Data from Microstim of a occlusion-positive afferent. Plot of normalized uEPSC amplitudes against trial number. The uEPSC data was obtained in the absence of HFS-STN (Control and Recovery) and after pairing with trains of HFS-STN (HFS-STN-conditioning). B) Same but occlusion test negative. C–F) Quantification of results. The test responses paired with HFS-STN underwent a marked depression of release, as indicated by reduced uEPSC amplitudes (C), reduced success rates (D), enhanced paired pulse ratios (E) and increased coefficients of variation (F). Note stronger effects in occlusion-positive afferents. The asterisks refer to the significance levels for differences with respect to the control values (ANOVA). The differences between Control and respective Recovery values were not significant and are omitted in the graphs, for clarity.

Fig. 3 presents the results obtained from a total of 9 occlusion-positive and 16 occlusion-negative STN afferents. It became clear that 100 ms after the conditioning HFS at half-effective stimulus intensity neither type of single fiber response has achieved full recovery (Fig. 3A, B). The calculated percent changes are summarized in Table 1. While a reduction of uEPSC amplitude (Fig. 3C) could be caused by a pre- as well as a postsynaptic process, alterations in the success rate (D), PPR (E) and CV (F) are better reconciled with a presynaptic site of action. Possible causes of depression might be excitability changes in the terminal arbour of the afferent axon, vesicle depletion and modification of calcium influx through G-protein coupled presynaptic receptors. The impact of these mechanisms could be different in occluded and non-occluded afferents, as the latter where not directly affected by the conditioning HFS-STN.

The examination of occlusion-negative afferents offers an opportunity to address non-intrinsic modulatory actions on release, e. g. effects derived from other HFS-STN-activated fibers that terminate in the neighbourhood of the tested axon. The plot obtained from the occlusion-negative afferent of Fig. 3B illustrates that the failure rate is now lower, but nonetheless amplitudes, success rates, PPR and CV were significantly altered in the population of occlusion-negative terminals (Fig. 3C–F, Table 1).

Assuming that the exocytosis of single vesicles is a probabilistic process, unitary test responses must exhibit a certain fraction of failures even though the active zone of transmitter release is successfully invaded by an AP. The presence of a postsynaptic response can be regarded as the best proof that the AP invaded the terminals. In multiquantal responses (several vesicles released simultaneously), a presynaptic depression should then reveal itself by a decrease in amplitude and an increase in the PPR, which was the case (Table 1). In uEPSCs from occlusion-negative glutamatergic connections (after exclusion of failure traces), the amplitude reduction and PPR increase were still significant.

At this point we can conclude that the glutamatergic synaptic afferents to the SNr experience a strong and lasting depression at their presynaptic terminals. The depression is not entirely intrinsic. Additional afferents are likely to be involved.

### Heterosynaptic depression of synaptic glutamate release due to activation of GABA(B) receptors

With the amount of presynaptic HFS-STN-induced depression already quantified for unitary glutamatergic afferents, we are still left with numerous possibilities of how this suppression is implemented. We turned to one of the most obvious possible causes of depression of the subthalamo-nigral pathway, i.e. modification of synaptic glutamate release by GABA acting at presynaptic GABA(B) receptors [49]. If this mechanism applies, the depression should be decreased by the presence of CGP55845, a potent selective GABA(B) receptor antagonist. We therefore compared the HFS-STN-induced depression of uEPSCs in the absence and in the presence of CGP55845 (1 µM). Thus, the strength of presynaptic depression at glutamatergic terminals was estimated indirectly, by the amount of changes induced by the antagonist CGP55845. This approach of pharmacological disinhibition is already well established (for instance: [50], [51]). At the given concentration, CGP55845 was shown to inhibit GABA(B) receptor-mediated responses up to saturating GABA concentrations, its IC50 being 5 nM [52], [53].

**Figure 4:**
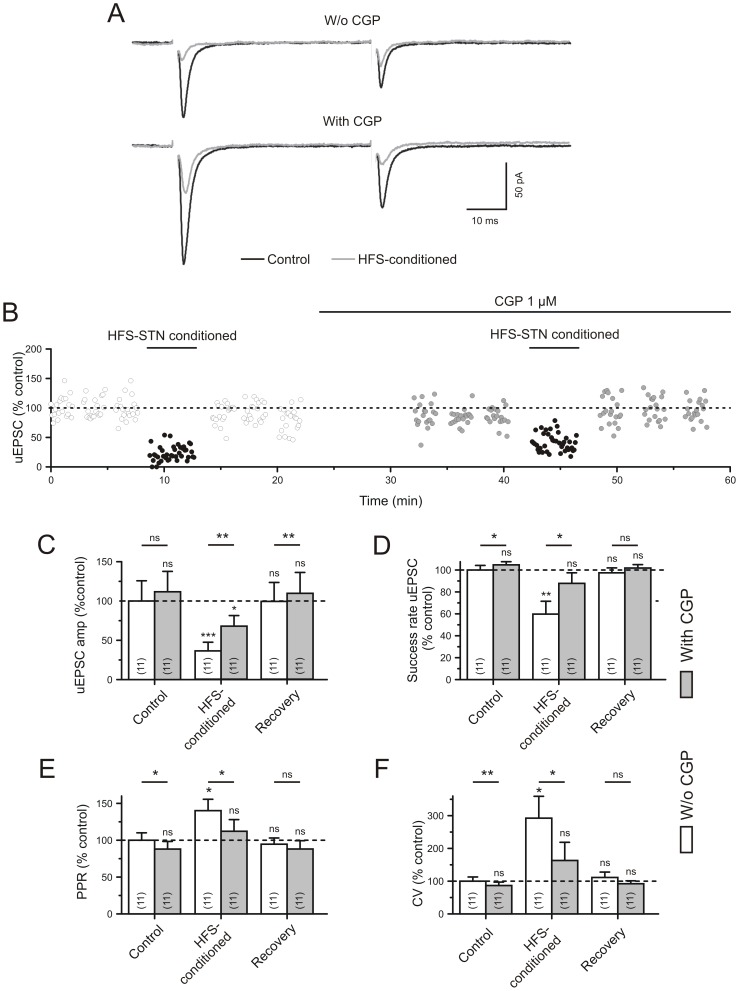
Block of GABA(B) receptors prevents the depressant effect of HFS-STN conditioning on unitary test responses induced by microstimulation of single STN axons in the SNr. A) Example traces to illustrate partial reversal of the HFS-STN effect on unitary EPSCs elicited with Microstim. Experiment in the presence of gabazine (15 µM). B) The plot of normalized uEPSC amplitudes against time of recording. The average value before the HFS trials is 100%. More than 10 min were allowed for the wash-in of the GABA(B) antagonist CGP55845 (1 µM). Note the alleviation of amplitude depression (B,C) and the increase in the success rate (D) in the presence of the GABA(B) blocker CGP55845. E,F) Quantification of the presynaptic parameters PPR and CV. The bars above the empty and shaded columns refer to the results of paired t-test. The asterisks above each column indicate significance levels from comparison with the Control, as tested with ANOVA. The differences between Control and respective Recovery values were not significant and are omitted in the graphs, for clarity.

The sample traces of Fig. 4A illustrate that GABA(B) receptor block with CGP prevented a significant amount of the HFS-STN-induced depression in subthalamo-nigral afferents. The plot of Fig. 4B shows one of 11 experiments performed in the same way. The quantification is given in Fig. 4C–F. It should be pointed out that in the absence of HFS-STN, i.e. under control conditions, GABA(B) receptor block had little effect. This indicates that at rest ambient GABA levels were not high enough to induce a significant tonic action at presynaptic GABA(B) receptors. In contrast, during HFS-STN ambient GABA levels significantly surpassed the threshold concentration needed to affect the presynaptic indicators PPR, success rate and CV. Only the uEPSC amplitudes did not fully recover suggesting that an additional postsynaptic process may be involved as well.

### HFS-STN liberates large amounts of GABA in the SNr

**Figure 5:**
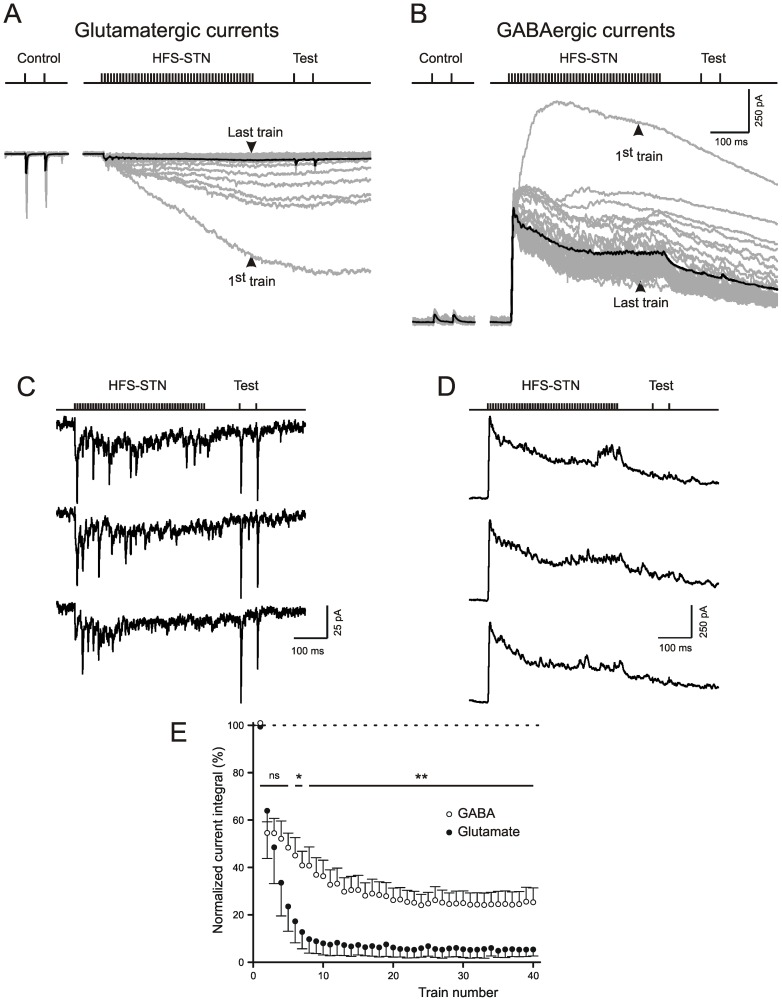
Compound glutamate- and GABA-induced ion currents during and after multi-unit HFS-STN. Responses of SNr neurons to Macrostim in the STN. Note that the driving force for the transmitter-induced currents is 60 mV for glutamate but only 50 mV for GABA. A) Recordings in the presence of gabazine (15 µM) to show compound glutamatergic ion currents. The calibration bars in (B) also apply to (A). Note strong attenuation with train repetition. B) Recordings in the presence of DNQX (10 µM) and APV (50 µM). Note that a fraction of the GABA-induced chloride current remains during and after HFS-STN. C,D) Enlarged individual traces from same experiments as in A,B, respectively. Shown are the last 3 trials of an HFS-STN conditioning session. The glutamatergic responses are strongly attenuated, GABAergic currents persist. E) Plot of current integrals during individual HFS-STN trials against trial number. The responses are normalized to the current integral during the first train (only cells with integral values >25 pA*s during first train included).

In the experiments described so far we have evaluated single-unit-induced uEPSCs as an indicator of changes produced by HFS-STN-conditioning. In the following series of experiments we refrained from single-unit activation, but instead recorded the compound current responses induced by HFS-STN itself (Fig. 5). Glutamatergic currents were isolated by the presence of the GABA(A) receptor blocker gabazine (Fig. 5A,C), and GABAergic currents were recorded in the presence of the glutamate receptor blockers DNQX and APV (Fig. 5B,D). In preceding experiments, the glutamatergic and GABAergic nature of the responses was determined by applying the appropriate antagonists at concentrations commonly used to block glutamatergic or GABAergic synaptic transmission. Note that the driving force of the two types of currents was slightly different (60 mV for glutamatergic and 50 mV for GABAergic currents). Nonetheless, the GABAergic currents were found to be substantially larger and, even more important, in part resisting the depressant action of HFS-STN (Fig. 5E).

These experiments demonstrate that a source of GABA is available at any moment during HFS-STN, due to synaptic release from GABAergic afferents.

### Unitary postsynaptic responses to single-unit HFS at GABAergic afferents

**Figure 6:**
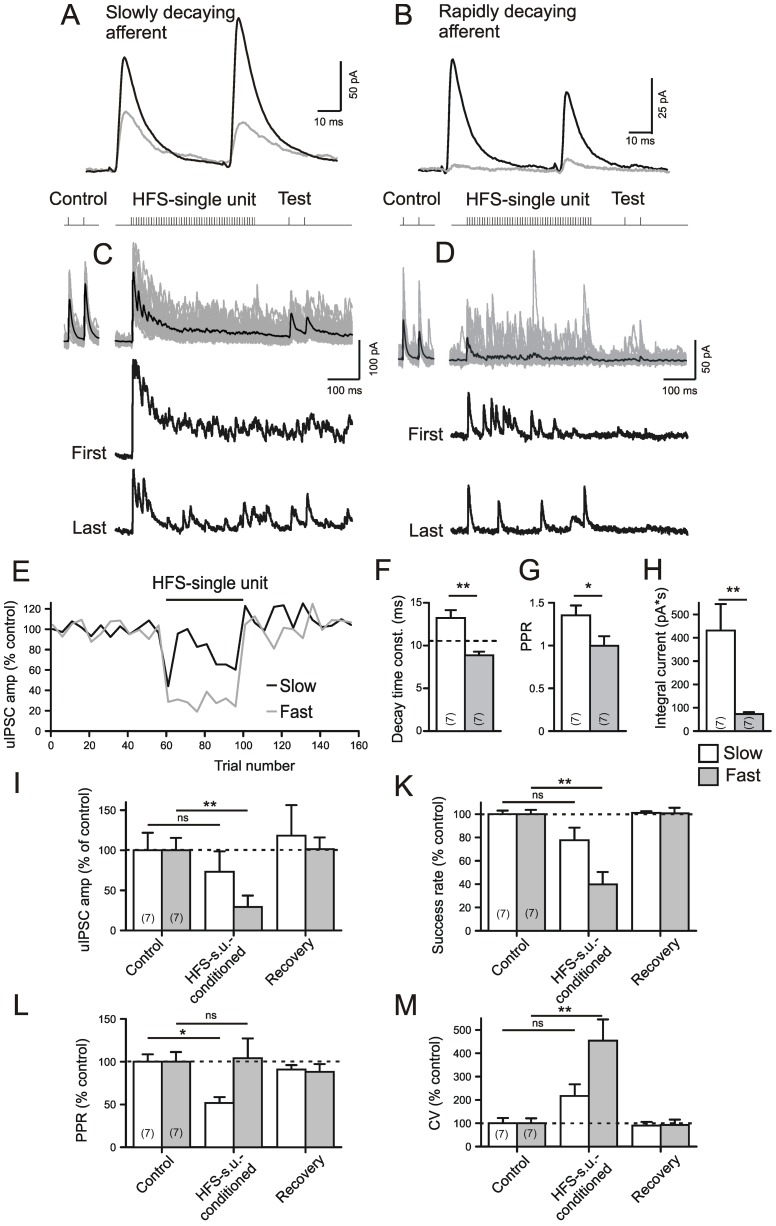
Two types of unitary IPSCs and their responses to single-unit HFS. A) Experiment in the presence of DNQX (10 µM) and APV (50 µM). Holding voltage −50 mV, chloride equilibrium potential −100 mV. A,B) Traces of averaged uIPSCs belonging to the more rapidly decaying type with paired pulse facilitation (A) and to the more slowly decaying type with paired pulse depression (B). Black trace: Control, Grey trace: HFS-conditioned. C, D) Single-unit HFS experiment. Traces belong to the same experiment as (A,B), respectively. Note larger integral current in C). E) Plot of normalized uIPSC amplitudes in response to the first test pulse following after HFS. Note persistence of current responses from afferents with slowly decaying uIPSCs. F–H) Characteristics of uIPSCs. Note differences in the time constant of decay (F), paired pulse ratio (G) and the integral current during HFS (H). The broken line in (F) denotes separation threshold. I–M) Quantification of results from HFS-conditioning. The differences between Control and respective Recovery values were not significant and are omitted in the graphs, for clarity.

Next we asked whether GABAergic afferents of different origin contributed equally to the persistent chloride conductance during and after HFS-STN. Again, the respective currents were pharmacologically isolated and kept at minimal intensity by threshold-toggling. Fig. 6A–D shows current traces of responses to single fiber stimulation of GABAergic afferents. One immediately notices the differences in the paired pulse plasticity (Fig. 6A, B and G), but there were also differences in the time constants of IPSC decay (Tau_Decay_).

Based on the differences in the Tau_Decay_ (Fig. 6F) we tentatively defined two types of afferents (“slow” and “fast”) and found significant differences in the PPR (Fig. 6G). Interestingly, the Tau-based grouping of afferents also predicted the behaviour during single-unit HFS. Specifically, units with slower decay depressed less in response to HFS (Fig. 6C, D, E) and the current integrals during HFS were significantly larger (Fig. 6H), and so was the degree of depression observed 100 ms after each HFS train (Fig. 6I–M).

Two previous studies on juvenile mice [37] and rats [36] suggested that the GABAergic inputs from the striatum and the *Globus pallidus* differ in their Tau_Decay_ and PPR values. To verify that Tau_Decay_ could be used as an identification criterion to the present fully adult slices from mice we performed additional experiments with minimal stimulation in the striatum. The resulting IPSCs were recorded under the same conditions as the uIPSCs elicited by single fiber activation in the SNr. The striato-nigral IPSCs had a mean Tau_Decay_ value of 11.4±1.5 ms (n = 14), and the mean PPR was 1.22±0.07 (n = 14). These values are not different from the means obtained from the “slow” group of afferents in Fig. 6. We therefore tentatively classified afferents with Tau_Decay_>11 ms and a PPR>1.2 as striato-nigral afferents.

Thus, a subclass of GABAergic afferents to the SNr retains release during single unit HFS and, most likely, these afferents originate in the striatum.

### Unitary postsynaptic responses to single-unit HFS at glutamatergic afferents

**Figure 7:**
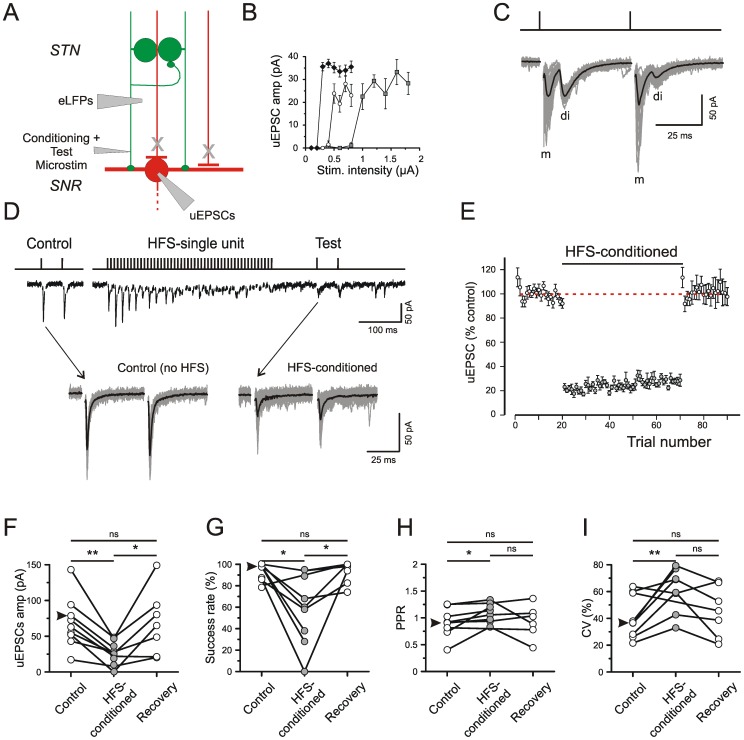
Dynamics of unitary EPSCs during single-unit HFS. A) Scheme of experiment. Note that in these experiments conditioning HFS and test pulse were applied to the same Microstim site. All recordings were performed in the presence of 15 µM gabazine. B) Estimation of threshold intensity for three single afferents to SNr neurons. C) Specimen records of responses to Microstim of a single STN fiber in the SNr. Note presence of monosynaptic (m) and disynaptic (di) uEPSCs. D) Single trial with traces with HFS and superposition of Control and HFS-conditioned traces from 20 trials. E) Plot of unitary test responses during Control (without HFS, 20 trials), HFS (50 trials) and Recovery (20 trials). Average and SE from 7 cells. F–I) Quantification of results. Arrow head denotes data from experiment illustrated in D.

Finally, single-unit HFS was also applied to glutamatergic afferents. This offered an opportunity to clarify the intrinsic depressant mechanisms in glutamatergic afferents (Table 1). Fig. 7A provides the scheme of experiment, and Fig. 7B presents three cases of minimal stimulation to activate glutamatergic unitary responses in the SNr. Typical traces of monosynaptic uEPSCs are shown in the bottom of Fig. 7D. However, in about one fifth of the cases (8/51) the first monosynaptic uEPSC was followed by a second disynaptic one (Fig. 7C). Here we only regarded the results obtained for monosynaptic uEPSCs, their latency ranging from 2 to 6 ms.

In the experiment of Fig. 7D we examined the behaviour of uEPSCs during low and high-frequency activation at the same site. 100 ms after the train, when FVs were already restored, uEPSCs were still depressed. This depression persisted throughout the HFS-part of the recording session (Fig. 7E), but recovery occurred in less than 6 s. Fig. 7E presents the mean ± S.E.M. for the uEPSC amplitudes normalized to the control average (n = 7). The results from individual glutamatergic afferents are given in Fig. 7F–I. Without exception, amplitudes were decreased and coefficients of variation were increased by preceding single-fiber HFS.

Comparing the amount of depression obtained with single-unit and multi-unit HFS in occlusion-positive afferents showed an apparent difference of the mean reduction of about 20%. However, with the relatively small number of tested afferents in each case this difference did not reach significance. We conclude that intrinsic depressant mechanisms are also present in the glutamatergic afferents.

## Discussion

**Figure 8:**
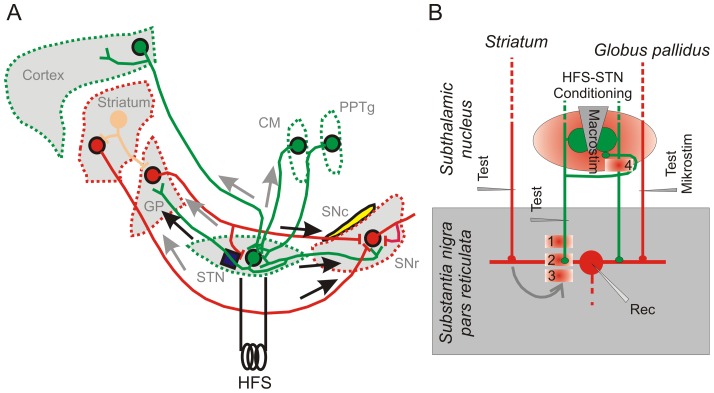
Hypothetical mechanisms of DBS effects. A) Simplified scheme of the neural connections presumably activated by HFS-STN in the rodent brain. Note that most of the collaterals and the entopeduncular nucleus were omitted. The subthalamic nucleus and the substantia nigra are shown enlarged. Abbreviations: GP - Globus pallidus, STN - subthalamic nucleus, SNr - Substantia nigra pars reticulata, SNc - Substantia nigra pars compacta. CM - centromedian nucleus, PPTg - Pedunculopontine tegmental nucleus. Ach - acetylcholine, DA - dopamine. For more details see [77]. The equivalents of primate and rodent structures are reviewed in [78]. B) Scheme of modifications presumably initiated by HFS-STN in the normal rodent brain. It is assumed that HFS-STN co-activates at least three types of afferents innervating the SNr: GABAergic afferents of striatal and pallidal origin and glutamatergic afferents of subthalamic origin. The latter is the core element of the presented circuit and exhibits the following four modifications: 1) Suppression of AP generation and conduction (recovery time: <100 ms, B) depletion of transmitter release (recovery time: <5 s), 3) heterosynaptic depression via presynaptic GABA(B) receptors (recovery time: <1 min), 4) depression of the recurrent pathway through the STN (recovery time: >10 min). Mechanism #1 has been studied in more detail by [35]. The present experiments focused on the mechanism #2. Details on mechanisms #3 and #4 will be published in a forthcoming paper.

Fig. 8 provides a highly simplified scheme of the neuronal connections through the basal ganglia focussing on the orthodromic and antidromic signal flow expected after electrical stimulation of the STN. Our present results can be summarized as follows. (i) The high frequency stimuli were applied at intermediate intensities, i.e. at intensities corresponding to the EC50 of single FVs recorded at the rostral pole of the SNr after bipolar STN stimulation via a metal electrode in the center of the STN. (ii) HFS-STN generates large compound glutamatergic and GABAergic currents in SNr neurons. The former undergo massive depression while part of the latter persists during repeated and/or prolonged HFS-STN trials. (iii) Pairing HFS-STN with glutamatergic unitary test responses revealed in the latter a major synaptic depression at a time when, according to FV data, AP generation is already restored (100 ms after the HFS-STN train). (iv) Depression was present in occlusion-positive units, i.e. in units electrically back-traced into the fiber pool activated by the conditioning STN macroelectrode (EPSC amplitude change: −74%). However, occlusion-negative afferents were also depressed (−57%). The latter finding points to an influence of co-activated afferents in the target area. (v) Exposing single glutamatergic and GABAergic afferents to HFS confirms the dominance of depression in the glutamatergic connections and reveals differential effects in GABAergic connections. (vi) Two classes of GABAergic inputs were separated according to their decay kinetics and paired pulse ratio. While the presumed pallido-nigral unitary connections exhibited complete depression, presumed striato-nigral afferents persisted in their activity and were able to provide GABA during and after HFS-STN. (vii) The HFS-STN-induced depression of synaptic glutamate release in the SNr was prohibited by block of GABA(B) receptors with CGP55845 (1 µM). The results suggest that the presynaptic depression of the glutamatergic subthalamo-nigral pathway is caused by GABA delivered by co-activated striato-nigral afferents and acting at presynaptic GABA(B) receptors.

### The present data in the light of previous results from other groups

As already revealed by other studies in rodents [23], HFS-STN elicited a mixture of excitatory and inhibitory responses in the SNr. Measurement of transmitter concentrations in the rat SNr showed elevation of glutamate and GABA concentration in dialysate samples obtained during and after HFS-STN [54], [55]. Lesion experiments suggested that the GABA-elevation observed at low stimulation intensities comes from GABAergic afferents originating in the *Globus pallidus* [56].

Deniau and colleagues [26] forwarded the idea that the beneficial effect of HFS-STN may reflect a gain of synaptic function and represent a condition where the excitation-inhibition balance is dramatically shifted as a consequence of differential sensitivity of excitatory and inhibitory afferents to HFS-STN. Our present results support and extend this hypothesis by presenting more direct evidence for a preferential suppression of the subthalamo-nigral and presumed pallidal-nigral afferents and the persistent activation of presumed striato-pallidal input.

We suppose that the relative robustness of the presumed striato-nigral transmission is due to the lower, in comparison with pallidal afferents, exhaustibility of the vesicle pool. Based on these results one can assume that the most effective way to facilitate a dominance of the direct as opposed to the indirect pathway would be to use intermediate current intensities that stimulate not only the axons of the subthalamo-nigral and the pallido-nigral neurons in the STN itself, but also the striato-nigral afferents travelling next to the STN [23].

This gain of function hypothesis stands somewhat in contrast with the fact that the pharmacologically isolated FVs recorded in our experiments and by [35], exhibit complete depression at intensities compatible with ongoing activity in the striato-nigral pathway. The most likely explanation is that at recording positions used to maximise the FVs in response to STN stimulation the striato-nigral fibers are too far away to substantially contribute to the HFS-STN-induced changes. Detailed FV mapping in whole animals is needed to further clarify this point.

### Possible mechanisms of synaptic depression in the subthalamo-nigral pathway

Our present study focused on the mechanisms available to reduce the excitatory synaptic input towards the SNr. Therefore we have used FV recordings mainly to establish a checkpoint for evaluation of synaptic activity independently on suppression in the main axon. Another approach was the demonstration of HFS-STN-induced depression in occlusion-negative unitary STN afferents with proven AP invasion into synaptic terminals (i.e. after exclusion of failure trials). This allowed us to reveal two types of mechanisms, i) an intrinsic depression present in any unit that experiences single fiber HFS and ii) a mechanism that requires participation of additional elements (heterosynaptic depression).

While a reduction of PSC amplitude can reflect pre- and postsynaptic parameters, the paired pulse ratio is often used to evaluate the state of the release machinery [40], [57]. It should be recalled that an increase in the paired pulse ratio is only possible when the vesicle pool is not fully depleted and reserve vesicles are available to produce a larger second response. If the readily releasable pool is depleted, one cannot expect that lower release probability with the first response will save vesicles for the 2^nd^ response. With the present day information on the dynamics of transmitter release in mind [58]–[61], we interpret the increase in the PPR in HFS-conditioned glutamatergic connections as evidence for a presynaptic depression in terminals with at least partial recovered vesicle pool. This depression can be strengthened by the release of GABA in the neighbourhood of the glutamatergic subthalamo-nigral terminals. If the concentration of perisynaptic GABA is high enough they would cause heterosynaptic depression via G-protein coupled presynaptic GABA(B) receptors.

The abundance of GABA(B) receptors in the SNr is well documented [62] and a depressant effect of GABA(B) receptors on subthalamo-nigral EPSCs has already been thoroughly quantified by Shen and Johnson [49]. In their slices from juvenile rats, baclofen at the highest concentration tested (10 µM) reversibly decreased EPSC amplitudes to 20% of the control. This result indicated that GABA(B)-mediated presynaptic depression is a powerful mechanism in control of glutamatergic input to the SNr.

Immunogold labelling for GABAB1 and GABAB2 was demonstrated on the presynaptic membrane of asymmetric, putative glutamatergic synapses. Presynaptic GABA(B) receptors reduce the Ca influx through voltage-activated channels [63], [64] and thereby decrease the probability of transmitter release [65]. Recent evidence from the Calyx of Held [66] suggests that the GABA-induced reduction of Ca influx not only leads to a lower release probability (which may reveal itself as an increase in the PPR) but also to a decrease in the size of the readily releasable vesicle pool, as detected by HFS at 100/s. It appears very likely that vesicle depletion and afferent-specific differences in the regulation of the vesicle pool play a role in HFS-STN.

As for the source of GABA responsible for the HFS-STN-induced depression of GABA release, our data in combination with the results of [36], [37] suggest that the non-depressing afferents were the striato-nigral afferents. Our present Tau_Decay_ values apply to recordings with zero chloride in the pipette, a condition known to prolong the decay [67]. The values obtained here under similar recording conditions with striatal stimulation were similar to those of the “slow” and HFS-STN resistant class of afferents, supporting the conclusion that the striato-nigral afferents represent a continuous source of GABA during HFS-STN.

A question deserving further attention concerns the possible role of postsynaptic receptor desensitization in the depression of glutamatergic afferents. The work of Windels and colleagues suggested that the glutamate elevation in the extracellular space outlasts the HFS-STN [56]. Although in the SNr block of GluR desensitization with cylothiazide was found to make no difference for the HFS-STN induced depression [13], the present experiments showing recovery of PPR but not amplitudes under condition of GABA(B) receptor block are consistent with a postsynaptic mechanism in depression. The alternative would have been a depression via presynaptic mGluR1 [68] or other modulators of presynaptic function such as dopamine [69] or adenosine [49]. However, presynaptic recovery by block of GABA(B) receptors was too complete to leave much range for another presynaptic depressant effect.

### Functional considerations and limitations of the current approach

As already mentioned in the introduction, animal studies on akinesia and its treatment with HFS-STN pursue three main directions [1], [2], [2]–[4], [26], [70]–[72]: A) Mechanisms preserving or recovering residual dopamine function, B) Mechanisms that restore normal cortical control including studies on the suppression of abnormal beta-oscillations and C) mechanisms facilitating the tonic inhibition in the output nuclei of the basal ganglia as a prerequisite of movement initiation. While mechanisms A) and B) have already been submitted to detailed analysis in dopamine-deficient rodents, the mechanisms of C) require additional work in animal models of akinesia or hypokinesia. What is becoming increasingly clear, however, is that the “activation” part of HFS is more important than initially envisioned. Although the results confirm a role of AP depression, we show that GABA release remains functional during HFS-STN, which most likely reflects the ongoing activity of the direct striato-nigral pathway.

In any system where HFS co-activates two or more types of afferents, the final outcome of repetitive fiber activation will depend i) on the relative robustness of release from a given unit and ii) on the number of afferents reaching the target area. Our slice preparation was optimized to preserve a maximum of the basal ganglia network. In any given slice the intactness of the BG connectome was verified by recording nigral evoked LFPs in response to cortical, striatal and pallidal stimulation. We believe that about 15 to 20% of the respective rostro-caudal connections are retained in 400 µm off-sagittal slices, if compared to the whole brain preparation (see Dvorzhak et al 2013, for a more detailed description of the slice preparation used here). This estimate is based on the fraction of cells that exhibit disynaptic EPSCs due to the activation of the recurrent trans-STN pathway and on the fraction of single fiber responses exhibiting occlusion by a preceding pulse through the subthalamic macro-electrode. The fact that among the striatal, pallidal and subthalamic afferents those of the most distal origin dominate the transmitter release in the SNr argues against fiber loss as a main cause of the shift of the glutamate/GABA balance towards lower levels of glutamate release.

The reductionist approach taken in the present study naturally favors the isolation of particular connections or mechanisms. Further studies will show to what extent the present conclusions derived from the analysis of single fiber responses will be applicable to the more complex situation in the intact brain. It should also be noted that there might be species-dependent differences in the structural organization of the BG in general and the STN-SNr pathway in particular. Specifically, there is no final clarity with regard to the contribution that local recurrent collaterals of STN neurons can make to the compound response to STN stimulation in primates [73]. In rodents we have such collaterals [74] and the strong depression of the disynaptic recurrent response has a major impact on the overall degree of depression in the STN-SNr pathway (Dvorzhak & Grantyn, unpublished).

Another issue to be considered is that clinical HFS-STN is mostly applied to individuals suffering from Idiopathic Parkinson Syndrome (IPS), i.e. degeneration of dopaminergic neurons in the *substantia nigra pars compacta (SNc)*. A low level of ambient dopamine may affect the glutamate/GABA balance in the SNr. Therefore, the present results should not be generalized to the situation in Parkinsonian mice. The animals studied here were WT siblings of z-Q175-KI homozygotes, a new mouse model of Huntington's disease. At the age of >1 year of life these mice exhibit a marked hypokinesia [75] along with a massive reduction in striatal dopamine release, with little if any decrease in the number of dopaminergic neurons in the SNc (Rothe, Stark, Paul, Dvorzhak & Grantyn, unpublished data from nonanesthetized homozygotes/WT). It remains to be determined whether dopamine-deficient z-Q175-KI homozygotes exhibit pathological burst activity in the *globus pallidu*s or STN and whether they generate enhanced oscillatory beta band activity, as it has been described for rodent toxin models of IPS (see for instance [11]). In any case, there might be dopamine-dependent differences in the plasticity of the subthalamo-nigral pathway, particularly in the GABA(B) mechanism of depression. Recent results from the striatum suggest that z-Q175-KI homozygotes exhibit a reduced tonic GABA(B)-mediated depression due to reduced nonsynaptic GABA release from astrocytes [76], while extracellular glutamate concentrations are elevated inducing a suppression of synaptic GABA release through an mGluR5/CB1 signaling cascade [41].

Despite of these caveats, the identification of heterosynaptic GABA(B)-dependent depression in the SNr is an essential step towards a better understanding of the highly complex cellular interactions associated with HFS-STN.
